# PM_2.5_ Spatiotemporal Variations and the Relationship with Meteorological Factors during 2013-2014 in Beijing, China

**DOI:** 10.1371/journal.pone.0141642

**Published:** 2015-11-03

**Authors:** Fangfang Huang, Xia Li, Chao Wang, Qin Xu, Wei Wang, Yanxia Luo, Lixin Tao, Qi Gao, Jin Guo, Sipeng Chen, Kai Cao, Long Liu, Ni Gao, Xiangtong Liu, Kun Yang, Aoshuang Yan, Xiuhua Guo

**Affiliations:** 1 Department of Epidemiology and Health Statistics, School of Public Health, Capital Medical University, Beijing, China; 2 Beijing Municipal Key Laboratory of Clinical Epidemiology, Beijing, China; 3 Graduate Entry Medical School, University of Limerick, Limerick, Ireland; 4 School of Medical Sciences, Edith Cowan University, Perth, Australia; 5 Beijing Municipal Science and Technology Commission, Beijing, China; The Ohio State University, UNITED STATES

## Abstract

**Objective:**

Limited information is available regarding spatiotemporal variations of particles with median aerodynamic diameter < 2.5 μm (PM_2.5_) at high resolutions, and their relationships with meteorological factors in Beijing, China. This study aimed to detect spatiotemporal change patterns of PM_2.5_ from August 2013 to July 2014 in Beijing, and to assess the relationship between PM_2.5_ and meteorological factors.

**Methods:**

Daily and hourly PM_2.5_ data from the Beijing Environmental Protection Bureau (BJEPB) were analyzed separately. Ordinary kriging (OK) interpolation, time-series graphs, Spearman correlation coefficient and coefficient of divergence (COD) were used to describe the spatiotemporal variations of PM_2.5_. The Kruskal-Wallis H test, Bonferroni correction, and Mann-Whitney U test were used to assess differences in PM_2.5_ levels associated with spatial and temporal factors including season, region, daytime and day of week. Relationships between daily PM_2.5_ and meteorological variables were analyzed using the generalized additive mixed model (GAMM).

**Results:**

Annual mean and median of PM_2.5_ concentrations were 88.07 μg/m^3^ and 71.00 μg/m^3^, respectively, from August 2013 to July 2014. PM_2.5_ concentration was significantly higher in winter (*P* < 0.0083) and in the southern part of the city (*P* < 0.0167). Day to day variation of PM_2.5_ showed a long-term trend of fluctuations, with 2–6 peaks each month. PM_2.5_ concentration was significantly higher in the night than day (*P* < 0.0167). Meteorological factors were associated with daily PM_2.5_ concentration using the GAMM model (*R* ^2^ = 0.59, AIC = 7373.84).

**Conclusion:**

PM_2.5_ pollution in Beijing shows strong spatiotemporal variations. Meteorological factors influence the PM_2.5_ concentration with certain patterns. Generally, prior day wind speed, sunlight hours and precipitation are negatively correlated with PM_2.5_, whereas relative humidity and air pressure three days earlier are positively correlated with PM_2.5_.

## Introduction

Ambient air pollutants, especially particulate matter (PM), have attracted attention in recent years because their associated adverse health effects [1–8]. It has been established that long- and short-term exposure to PM, including particles with a median aerodynamic diameter < 2.5 μm (PM_2.5_) and < 10 μm (PM_10_), elevates the risk of cardiovascular and respiratory diseases and excess mortality [1–3]. Research suggests that PM_2.5_ is very toxic and more harmful to human health than coarse particles (particles with a median aerodynamic diameter > 2.5 μm). When inhaled, PM_2.5_ enters the bloodstream and translocated to vital organs including the liver, spleen, heart and the brain [9]. Adverse health outcomes from PM_2.5_ inhalation include, among others: impaired pulmonary function, increased blood pressure, and cognitive deficit [4–6]. PM_2.5_ can also lead to stroke, lung cancer, and some other illnesses [7, 8].

China has experienced rapid urbanization and industrialization, which has resulted in a dramatic increase in energy consumption and emission over the past several decades [10]. One of the environmental challenges is the frequent nationwide episodes of haze-fog. A recent study reported that the annual average concentration of PM_2.5_ for almost all provincial capital cities in China exceeded 35 μg/m^3^ during 2013–2014 [11, 12]. It appears that the threat is more serious in the capital city, Beijing, China, in part due to its large population size, increase number of vehicles and numerous active construction activities. For example, during 2004–2008, daily mean PM_2.5_ concentration was 105 μg/m^3^, and the latest study revealed that citywide cumulative number of exceedance days is generally high [13, 14]. The extremely high concentrations of PM_2.5_ can lead to various negative health outcomes, several studies have shown that PM_2.5_ has significant effects on cardiovascular and respiratory emergency room visits, as well as years of life lost in Beijing [13, 15, 16].

Considering the multiple deleterious health effects of PM_2.5_, data with high spatial and temporal resolution are needed to accurately evaluate the status and health risks associated with PM_2.5_ exposure. However, access to pre-existing PM_2.5_ data from the Beijing Environmental Protection Bureau (BJEPB) has not possible since most of the PM_2.5_ data for the previous years were not documented. It was until October 2012 that the hourly monitoring data of PM_2.5_ was released. This data was sampled from 35 sites which is a representative of the whole city. Although the spatiotemporal distribution of PM_2.5_ using these data was reported in one study, continuous concentrations of PM_2.5_ at high temporal resolution were unavailable [14]. Other investigators reported long-term variation of PM_2.5_, but their results were generally based on discrete points or indirect estimation [17, 18]. Several studies have explored the relationship between meteorological factors and PM_2.5_ in Beijing and found that meteorological factors may be important in PM_2.5_ variation. However, only few of these studies have examined the correlation between wind speed and relative humidity and PM_2.5_. Additionally, most of these studies have not fully explored the impact of various meteorological variables on PM_2.5_ [19–21].

The purpose of the present study is to examine the spatiotemporal variations of PM_2.5_ in Beijing, using officially released data from 35 stations during a one-year period from August 2013 to July 2014, and to assess the relationships between daily PM_2.5_ and meteorological factors.

## Methods

### Source of PM_2.5_ and meteorological factors

**Fig 1:**
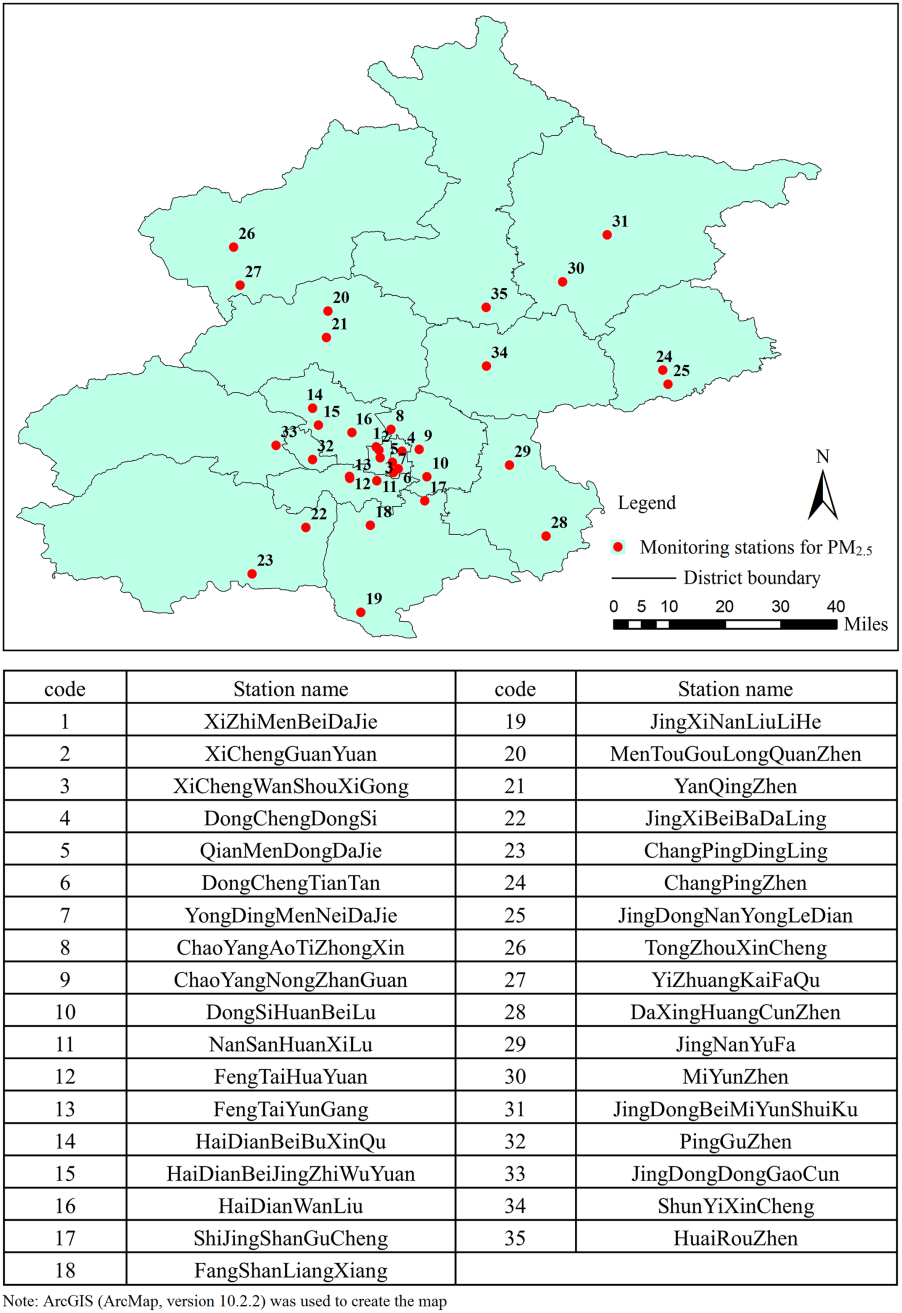
Locations of the 35 PM_2.5_ monitoring stations in Beijing.

Since the end of September 2012, daily average and hourly real-time ambient air pollutant data have been gradually released to the public by the BJEPB, based on the 35 automatic monitoring stations established in the 16 districts of Beijing city (Fig 1). Daily average (August 2013 through July 2014) and hourly real-time (December 2013 through November 2014) of PM_2.5_ concentration data were collected from the Centre of the City Environmental Protection Monitoring Website Platform, BJEPB (www.bjmemc.com.cn). In addition, meteorological data including daily mean wind speed (m/s), relative humidity (%), sunlight hours (h), temperature (°C), precipitation (mm) and air pressure (kPa) in the 16 districts were obtained from the Chinese Meteorological Bureau over the same period.

Daily mean concentrations for each district and the whole city were calculated by averaging concentrations reported by all 35 stations, which is the same method used by BJEPD to report daily concentration of air pollutants to the public. Rates of missing values in the 16 districts were mostly low ranging from 7.12% to 8.77%, except for *Mentougou* and *Huairou* which had higher levels of 17.81% and 10.41%, respectively. Some daily data were missing for all the districts mainly due to the technical problem on website maintenance during the study period. A Markov chain Monte Carlo (MCMC) multiple imputation method was used to impute missing values, and data from 339 days were available for analyses.

### Spatiotemporal analysis of PM_2.5_

To provide a more comprehensive picture of the current status and spatiotemporal variations of PM_2.5_ pollution, daily and hourly concentration data were analyzed by different methods. Using the Chinese ambient air quality standards (CAAQS) as a reference, daily average PM_2.5_ that exceeded Grades I (35 μg/m^3^) and II (75μg/m^3^) were selected.

Ordinary kriging (OK) interpolation [22, 23] was used to characterize PM_2.5_ regional and seasonal variations, based on concentration data from the 35 monitoring stations. PM_2.5_ summary statistics, space-time dependence functions and PM_2.5_ estimates on a space-time grid were obtained to describe regional and seasonal variations. This was done using the Geostatistical Analyst Extension of ArcGIS (ArcMap, version 10.2.2). To explore PM_2.5_ regional and seasonal variations, 16 districts were assigned to three areas: southern, northern and central (Table 1). Furthermore, 12 months were stratified into four seasons, spring (March, April and May), summer (June, July and August), autumn (September, October and November) and winter (December, January and February).

**Table 1:** Distribution of PM_2.5_ concentrations in the 16 districts of Beijing, 2013–2014.

District	Regional category	Mean(SD) μg/m^3^	Median (IQR) μg/m^3^	Range μg/m^3^	Non-attainment days and rates (%) for grade I	Non-attainment days and rates (%) for grade II
**Yanqing**	**North**	**67.95(61.06)**	**52.00(67.00)**	**5.0–459.0**	**216 (63.7)**	**108 (31.9)**
**Changping**	**North**	**73.30(66.36)**	**55.00(72.00)**	**5.0–432.0**	**224 (66.1)**	**130 (38.3)**
**Miyun**	**North**	**67.79(61.74)**	**51.00(68.00)**	**4.0–481.0**	**205 (60.5)**	**117 (34.5)**
**Huairou**	**North**	**77.80(65.80)**	**59.00(72.00)**	**5.0–402.0**	**226 (69.1)**	**136 (41.6)**
**Pinggu**	**North**	**79.23(66.25)**	**61.00(74.50)**	**4.0–475.0**	**242 (71.4)**	**150 (44.2)**
**Shunyi**	**North**	**83.55(70.27)**	**65.00(80.00)**	**5.0–509.0**	**241 (71.5)**	**149 (44.2)**
**Haidian**	**Center**	**85.15(70.47)**	**71.00(77.00)**	**4.0–430.0**	**248 (73.2)**	**161 (47.5)**
**Shijingshan**	**Center**	**87.99(69.32)**	**72.00(73.00)**	**6.0–408.0**	**254 (76.3)**	**158 (47.4)**
**Xicheng**	**Center**	**88.26(71.60)**	**70.50(78.00)**	**6.0–449.0**	**253 (74.6)**	**161 (47.5)**
**Chaoyang**	**Center**	**89.24(71.33)**	**71.50(81.00)**	**5.0–464.0**	**262 (77.3)**	**165 (48.7)**
**Dongcheng**	**Center**	**91.56(72.97)**	**75.00(81.00)**	**3.0–457.0**	**266 (78.5)**	**168 (49.6)**
**Fengtai**	**Center**	**96.50(76.59)**	**79.00(82.00)**	**6.0–511.0**	**268 (79.3)**	**184 (54.4)**
**Mentougou**	**South**	**77.41(64.88)**	**65.50(66.75)**	**5.0–403.0**	**211 (70.3)**	**124 (41.3)**
**Fangshan**	**South**	**106.97(79.33)**	**89.00(91.00)**	**6.0–492.0**	**285 (84.3)**	**194 (57.4)**
**Daxing**	**South**	**106.20(83.77)**	**86.00(93.50)**	**7.0–493.0**	**280 (82.6)**	**192 (56.6)**
**Tongzhou**	**South**	**107.63(85.87)**	**88.00(92.00)**	**4.0–537.0**	**287 (84.9)**	**193 (57.1)**

SD: standard deviation; IQR: inter-quartile range.

In addition, day to day variation of PM_2.5_ citywide during the year was displayed as a time-series figure. The number of PM_2.5_ pollution episodes (periods with concentrations > 75 μg/m^3^), episode duration, and interval between two episodes were calculated. Diurnal variations of PM_2.5_ in each month were developed into time-series figures by averaging the concentrations at various time points.

To assess PM_2.5_ spatial heterogeneity, Spearman correlation coefficients and coefficients of divergence (COD) were calculated for each monitoring station pair, and compared with the distance between the stations [24, 25]. A low COD value indicates small differences between stations PM_2.5_ concentrations, while a value close to 1 signifies greater disparity between concentrations.

Kruskal-Wallis H and Bonferroni correction tests were used to assess differences in PM_2.5_ levels associated with spatial and temporal factors, including season, area, and daytime. Weekday/weekend differences were tested by Mann-Whitney U test. All statistical tests were two-sided, and *P*-values less than 0.05 were considered statistically significant.

### Modeling association between PM_2.5_ and meteorological factors

Because scatter plots showed that not all meteorological variables were linearly correlated with PM_2.5_, a generalized additive mixed model (GAMM) was used to explore the effects of meteorological factors on daily PM_2.5_ concentrations. This model can use both additive parametric terms and nonparametric function to formulate covariate effects and add random effects to the additive predictor, accounting for over dispersion and correlation [26, 27]. District-level daily PM_2.5_ concentration data were used as the dependent variable, and corresponding district-level meteorological factors were used as independent variables. Lagged (1–3 days earlier) effects of meteorological factors were checked, because the prior weather conditions may influence the subsequent concentrations of PM_2.5_ [28]. Meteorological variables that had the strongest correlation with PM_2.5_ from lag0 (current value) to lag3 (value 3 days earlier) with Spearman correlation coefficient *r*_*s*_ > 0.2 were entered in the final model. The Akaike Information Criteria (AIC) and adjusted *R* ^*2*^ were used to select the appropriate variables and models.

The conditional probability distribution of PM_2.5_ concentrations approximately followed a Gamma distribution according to QQ plot and was tested by one-sample Kolmogorov-Smirnov test, so a logarithm-linked function for PM_2.5_ concentration was used in the GAMM model. Cubic splines were used as the nonparametric function of the covariates, which were potentially not linearly correlated to log-transformed PM_2.5_ [29]. Day of the year was introduced to control temporal effects on PM_2.5_ concentration. An automatic choice was adopted to determine the most appropriate parameters (degrees of freedom, knots) for the splines, based on generalized cross-validation (GCV). In addition, since PM_2.5_ concentration depends linearly on its own previous values and on a stochastic term, an autoregressive structure *ARMA(p*,*q)* was introduced in the model to describe the regression [30]. Optimal values of *p* and *q* were determined by AIC and autocorrelation function (ACF). The initial model is

$$\text{log}\left(E\left(Y_{i,t}\right)\right)=\alpha +s_{1}\left(Day_{i}\right)+s_{2}\left(WS_{i,t}\right)+s_{3}\left(RH_{i,t}\right)+s_{4}\left(T_{i,t}\right)+s_{5}\left(SH_{i,t}\right)+\lambda DOW\left(P_{i,t}\right)+\beta AP_{i,t}+\mu Z_{i}+\tau_{t}$$

Where *Y*_*i*,*t*_ is the concentration of PM_2.5_ in district *i* (*i* = 1 to 16) on day *t* (*t* = 1 to 339). Each *s* represents a cubic splines smoothing function for meteorological factors including wind speed (*WS*), relative humidity (*RH*), temperature (*T*) and sunlight hours (*SH*), which exhibit non-linear relationships with log-transformed daily PM_2.5_ concentration. *s*(*Day*_*i*_) was used to control for temporal trend. Since precipitation (*P*) followed an extreme skewed distribution and air pressure (*AP*) was linearly correlated with PM_2.5_, a dichotomous form of precipitation and linear term of air pressure were introduced in the model. *Z*_*i*_ is a random intercept for district *i* and *τ*_*t*_ is the autoregression term. All analyses were conducted using statistical software R (version 3.1.2), and package “mgcv” was used for the GAMM modeling. All statistical tests were two-sided, and *P*-values less than 0.05 were considered statistically significant.

## Results

### Overview of PM_2.5_ pollution in Beijing

Annual mean PM_2.5_ concentrations ranged from 67.79 μg/m^3^ in district *Miyun* to 107.63 μg/m^3^ in district *Tongzhou*, greatly exceeding the yearly CAAQS (GB3095-2012) Grade I (15 μg/m^3^) and II standards (35 μg/m^3^) for all districts in Beijing (Table 1). The citywide mean concentration of 88.07 μg/m^3^ also exceeded the standards. Table 1 lists the number of non-attainment days (defined as days with PM_2.5_ concentration exceeding standards) and rates for the 16 districts based on the daily CAAQS (GB3095-2012) standards. All the 16 districts experienced PM_2.5_ pollution that exceeded daily Grade I (35 μg/m^3^) standard during more than 60% of days (a non-attainment rate of 60%) and Grade II (75 ug/m^3^) standard during over 30% of days (a non-attainment rate of 30%) of the year.

### Spatiotemporal variations of PM_2.5_ pollution

**Fig 2:**
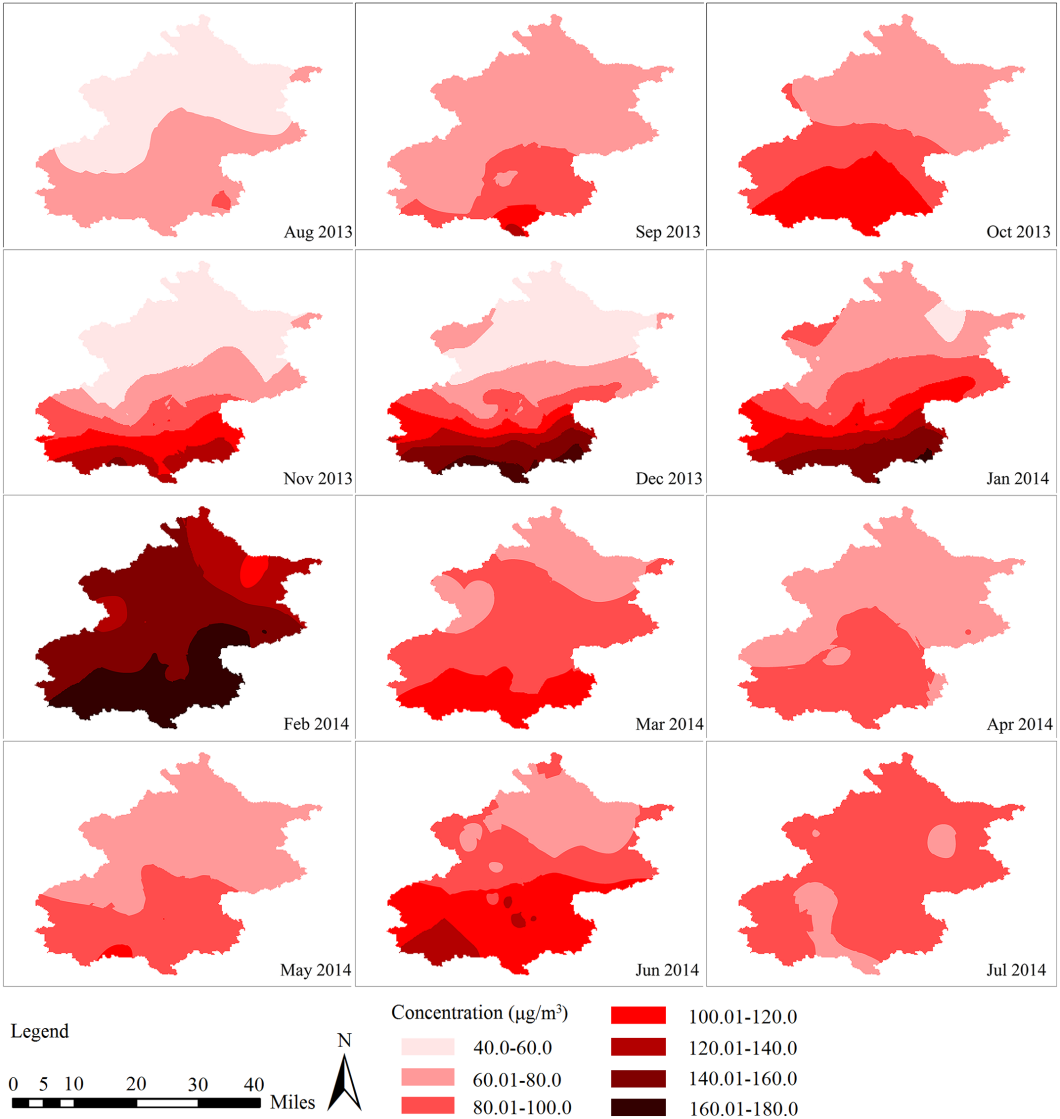
Regional and seasonal variations of PM_2.5_ in Beijing, 2013–2014.

Bonferroni test was used to assess seasonal and regional differences in PM_2.5_ levels, and the mean difference was significant at the 0.0083 and 0.0167 levels, respectively (Table 2). PM_2.5_ pollution in Beijing had pronounced seasonal and regional variations (Fig 2). It was significantly higher in winter (*P* < 0.0083) and lower in summer (*P* < 0.0083). There was no statistically significant difference in PM_2.5_ concentration between spring and autumn (*MD* = −5.615, *P* = 0.024). PM_2.5_ concentration in the southern part of the city was significantly higher than the northern area (*MD* = 29.492, *P* < 0.0167). Observed PM_2.5_ levels also revealed a pronounced spatial gradient, increasing from north to south in most months, except in July 2014. This pattern was more obvious in cold months (November 2013 to January 2014), with extremely high concentrations in the southern part of the Beijing city.

**Table 2:** Significance tests of PM_2.5_ levels for different season, region, daytime and day of week.

Variable		Kruskal-Wallis H test		Bonferroni test	
		*χ* ^2^	*P*	*MD*	*P*
**Season** *	**Spring vs. Summer**	**367.720**	**0.000**	**9.945**	**0.000**
	**Spring vs. Autumn**			**-5.615**	**0.024**
	**Spring vs. Winter**			**-38.140**	**0.000**
	**Summer vs. Autumn**			**-15.560**	**0.000**
	**Summer vs. Winter**			**-48.085**	**0.000**
	**Autumn vs. Winter**			**-32.525**	**0.000**
**Region** ^**#**^	**South vs. North**	**304.553**	**0.000**	**29.492**	**0.000**
	**North vs. Center**			**-16.254**	**0.000**
	**Center vs. South**			**-13.238**	**0.000**
**Daytime** ^**#**^	**7 pm-6 am vs. 7 am-12 am**	**69.991**	**0.000**	**12.839**	**0.001**
	**7 pm-6 am vs. 1 pm-6 pm**			**7.855**	**0.000**
	**7 am-12 am vs. 1 pm-6 pm**			**4.985**	**0.135**
				**Mann-Whitney U test**	
				***Z***	***P***
**Day of week**	**Weekdays vs. Weekends**			**-0.145**	**0.885**

*: The mean difference is significant at the 0.0083 level for Bonerroni test.

^#^: The mean difference is significant at the 0.0167 level for Bonerroni test.

**Fig 3:**
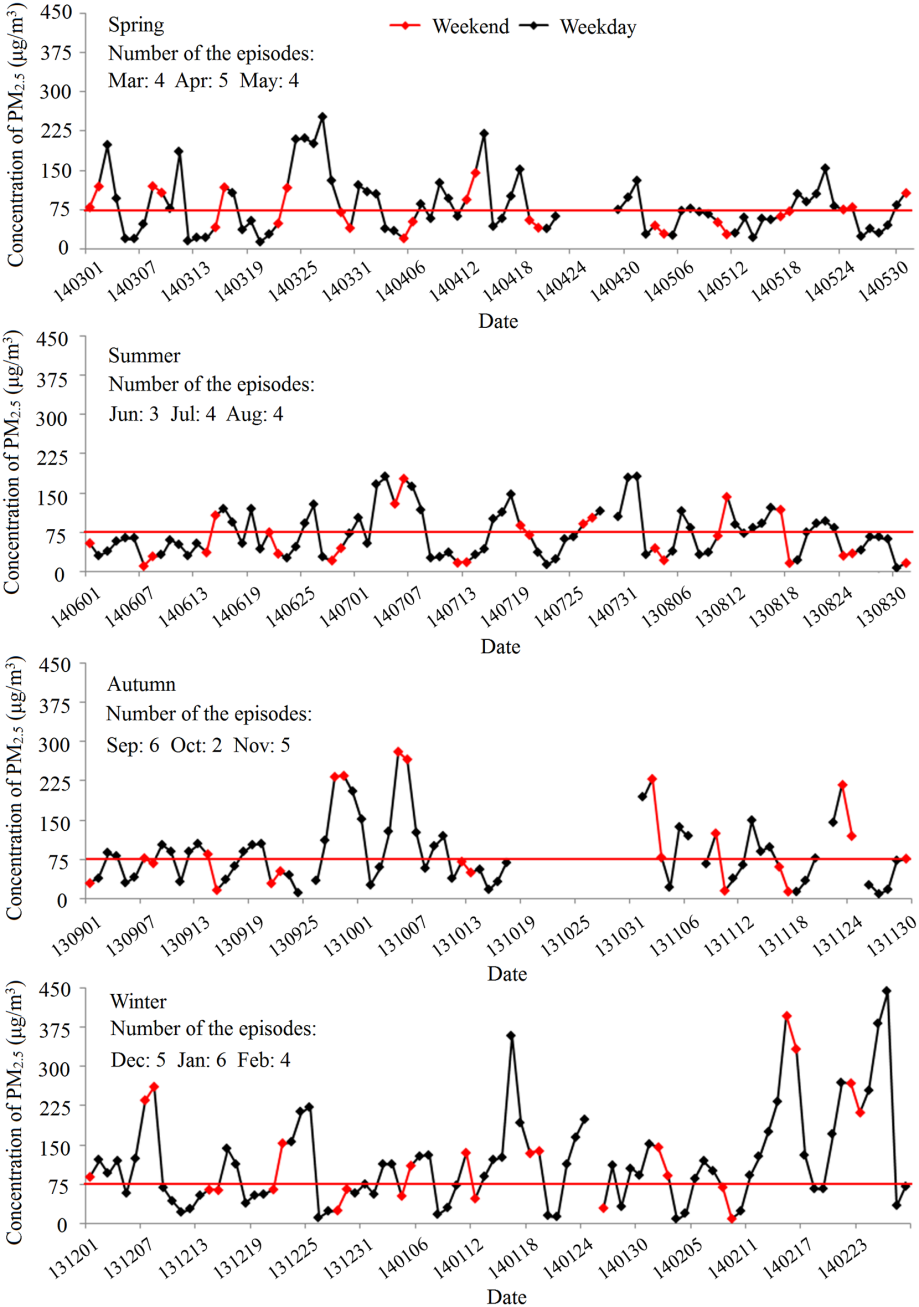
Day to day variations of PM_2.5_ in different seasons, Beijing, 2013–2014.

The day to day pattern of PM_2.5_ concentrations from August 2013 to July 2014 showed a long-term trend of fluctuations (Fig 3). A total of 52 episodes of PM_2.5_ pollution (> 75 μg/m^3^) were observed during the year (13 in spring, 11 in summer, 13 in autumn and 16 in winter) with 2–6 episodes each month. An episode usually lasted 1–7 days, and intervals between episodes were 1–14 days (missing days were not included in the calculation). Mann-Whitney U test was used to assess weekday/weekend difference, but no statistically significant difference was found (Fig 3 and Table 2) (*Z* = −0.145, *P* = 0.885).

**Fig 4:**
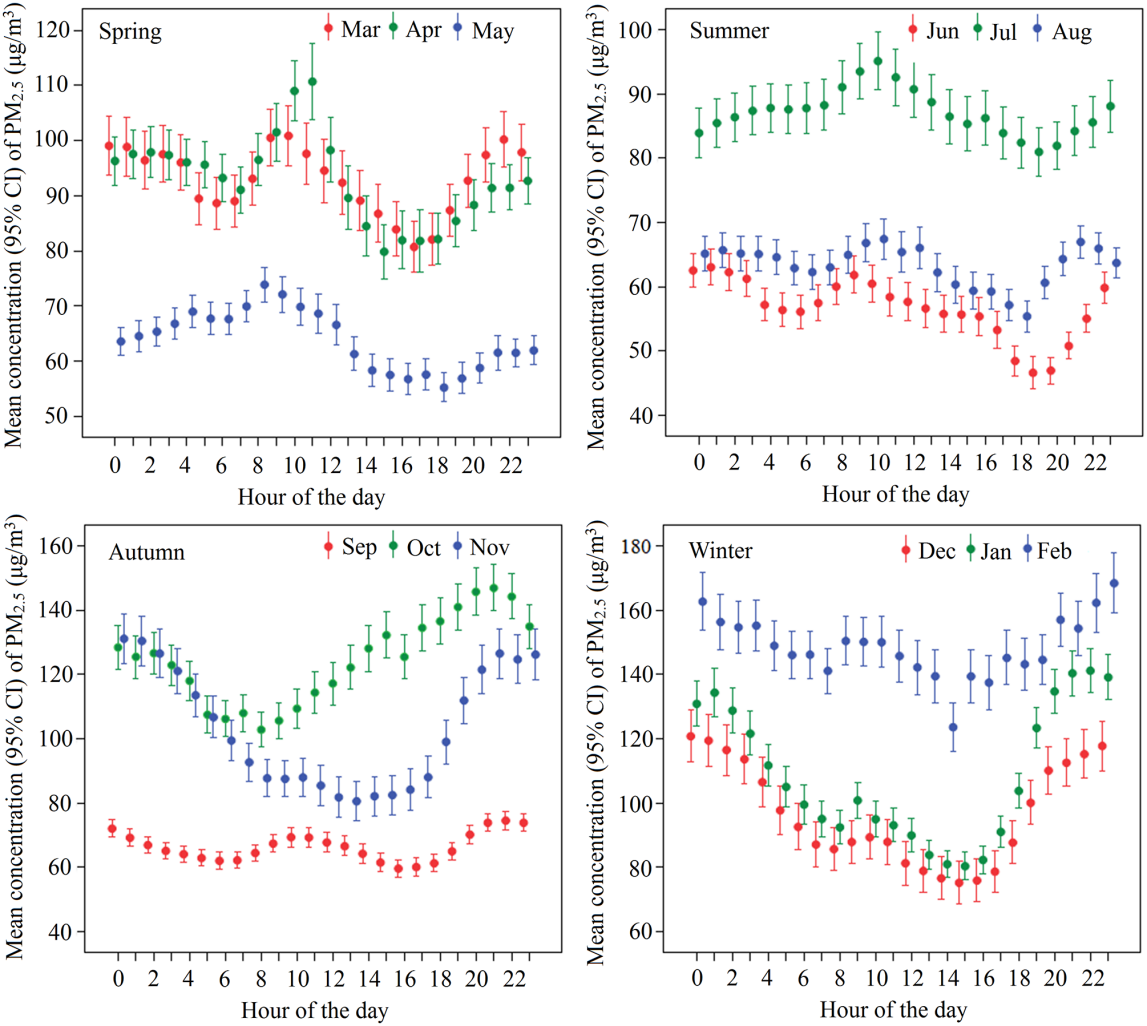
Diurnal variations of PM_2.5_ in different months, Beijing, 2013–2014.

Hourly average PM_2.5_ concentration in each month had a diurnal pattern represented by one or two peaks. For given days, if the PM_2.5_ concentration increased from a value lower than the monthly mean to one higher than that mean, those days were regarded as a single peak until the concentration fell below the mean (Fig 4). Over 7 months (February to April, June to September), there were 2 peaks, 1 in the forenoon, and the other in the early night time. For the other 5 month (May, and October to January), the peak was either in the forenoon or early night time. The lowest PM_2.5_ levels were in the afternoon, except during October. Bonferroni test was used to assess hourly difference in PM_2.5_ levels, and the mean difference was significant at the 0.0167 level (Table 2). It shows that PM_2.5_ concentration at night (7 pm through 6 am) was significantly higher than in the daytime (7 am through 12 am and 1pm through 6 pm) (*P* < 0.0167), but there were no statistically significant difference between forenoon (7 am through 12 am) and afternoon (1 pm through 6 pm) (*MD* = 4.985, *P* = 0.136).

**Fig 5:**
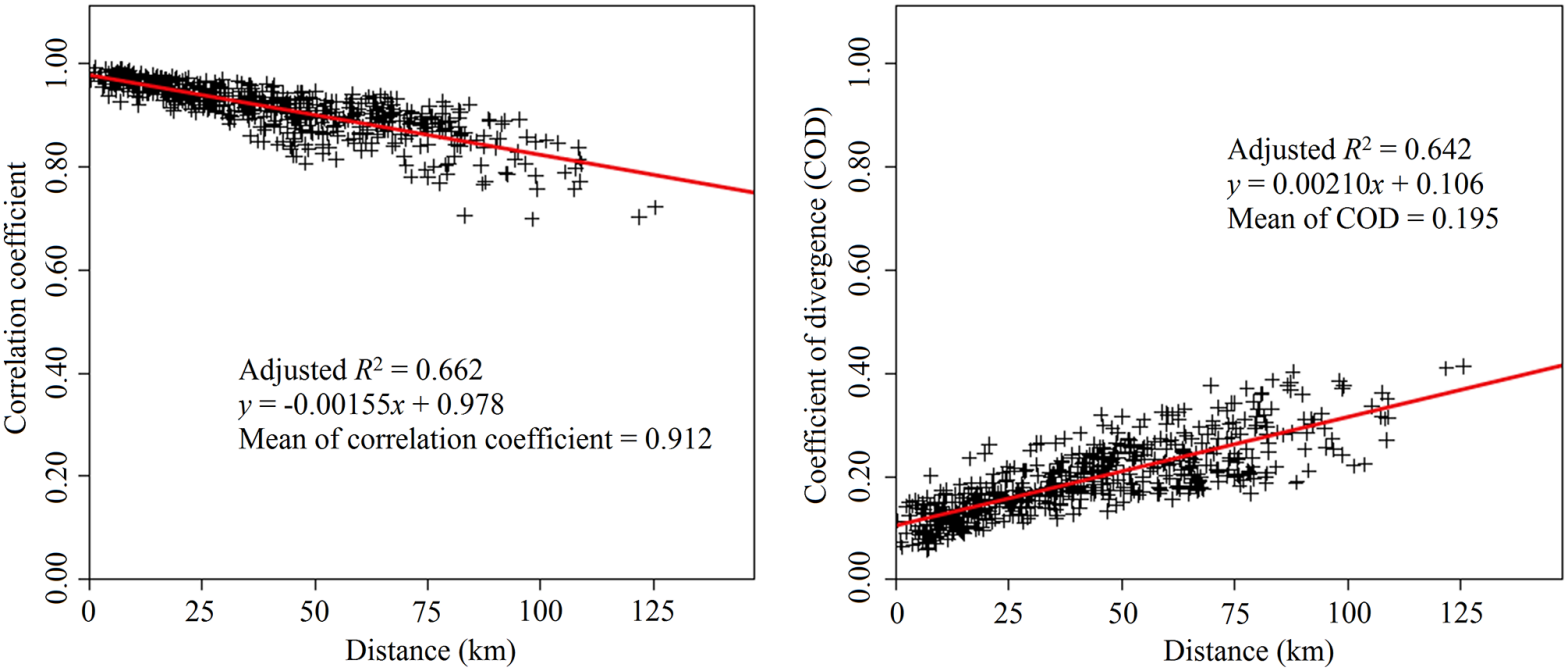
Correlation coefficient and COD versus distance between the stations.

The spatial heterogeneity of PM_2.5_ was examined by calculating correlation coefficients and CODs for daily average concentrations at 595 station pairs. Mean values of the two coefficients for all station pairs were 0.912 and 0.195, respectively (Fig 5). Fig 5 shows that correlation coefficients declined with increasing distance between stations, whereas CODs increased with increasing distance between stations. Slopes of both fit lines in Fig 5 were significantly different from zero (*P* < 0.05).

### Association between PM_2.5_ and meteorological factors

Correlation analysis showed that prior day wind speed (*r*_*s*_ = −0.48, *P* < 0.01) and air pressure 3 days earlier (*r*_*s*_ = 0.26, *P* < 0.01) were highly correlated with the current PM_2.5_ concentrations (S1 Table). For relative humidity (*r*_*s*_ = 0.38, *P* < 0.01) and sunlight hours (*r*_*s*_ = −0.51, *P* < 0.05), the strongest correlation was in the day of PM_2.5_ measurement. Because correlation coefficients of temperature (lag0 − lag3) were all < 0.2 at various daytimes, they were not included in the final model. For dichotomous variable precipitation, the model without a lagged term had the smallest AIC and largest adjusted *R* ^*2*^. Thus, meteorological variables including prior day wind speed (*WSlag1*), relative humidity (*RH*), sunlight hours (*SH*), precipitation (*P*) and air pressure 3 days earlier (*APlag3*) were entered in the final model. We selected the order of the autoregressive error term *p* = 2 and *q* = 2 with the smallest AIC, and the autocorrelation fall between [0.1, 0.1] from the ACF. The final model is

$$\text{log}\left(E\left(Y_{i,t}\right)\right)=\alpha +s_{1}\left(Day_{i}\right)+s_{2}\left(WSlag1_{i,t}\right)+s_{3}\left(RH_{i,t}\right)+s_{4}\left(SH_{i,t}\right)+\lambda DOW\left(P_{i,t}\right)+\beta APlag3_{i,t}+\mu Z_{i}+corARMA\left(2,2\right)$$

**Fig 6:**
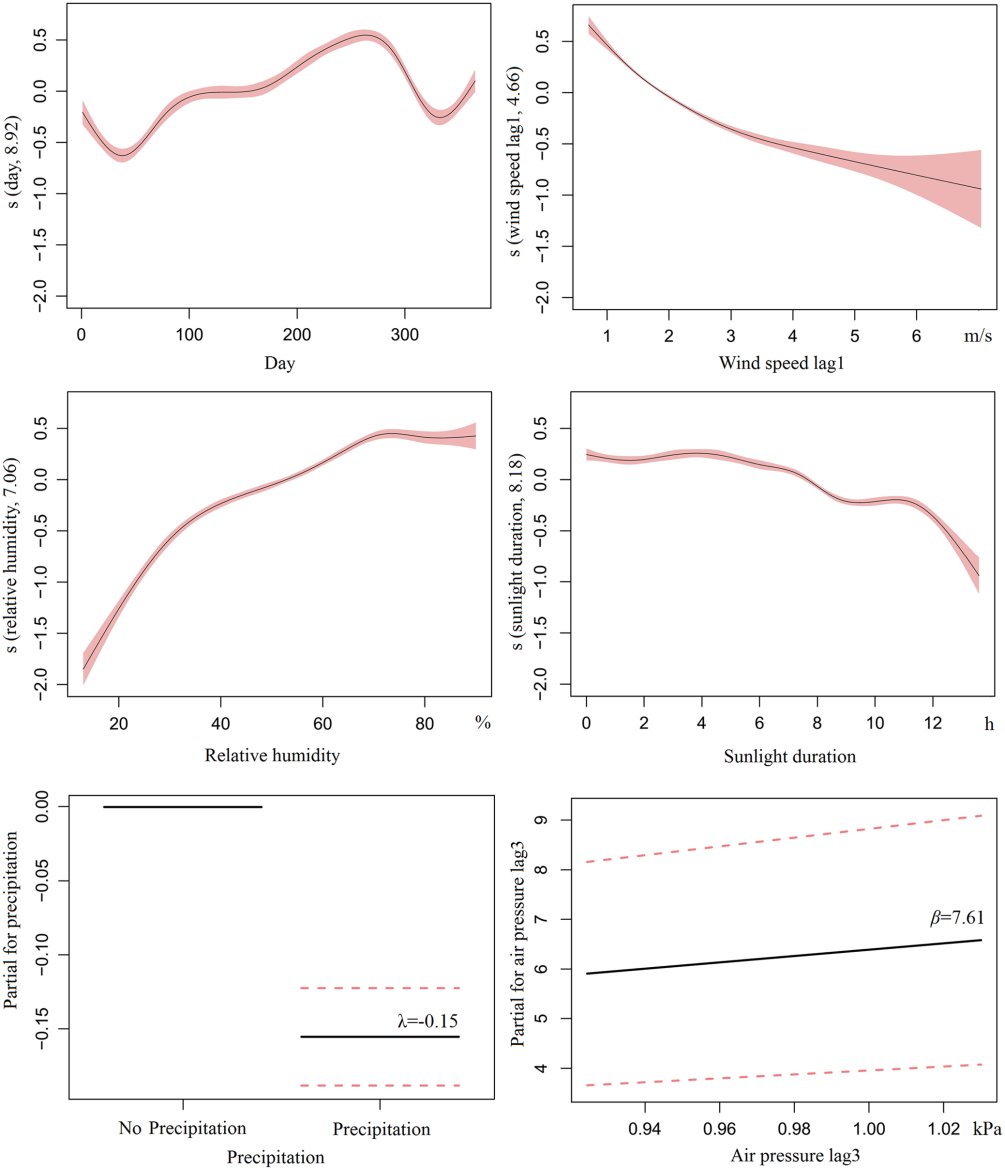
Exposure-response curves for PM_2.5_ and meteorological variables, Beijing, 2013–2014.

Overall effect size measured by the adjusted *R* ^2^ was 0.59 and goodness-of-fit assessed by the AIC was 7373.84 for the final GAMM model. The relationship between PM_2.5_ and prior day wind speed was monotonically decreasing (Fig 6). Similarly, an overall downward tendency was found for PM_2.5_ with increasing sunlight hours. On the contrary, PM_2.5_ was positively correlated to relative humidity. For the dichotomous precipitation variable, PM_2.5_ concentration was 85.68% (95% CI: 82.98%–88.47%) on days with precipitation, compared with those days of without precipitation. Air pressure had a 3-day lag effect on PM_2.5_, which was positively correlated with log-transformed PM_2.5_ concentration in linear from.

## Discussion

The study shows that Beijing has serious PM_2.5_ pollution citywide throughout the year [12–14]. We observed great spatial variations across the city [14, 31], with a pronounced increasing gradient from the north to the south. Southern Beijing is adjacent to seriously polluted cities in *Hebei* province and *Tianjin* [12, 32, 33]. Regional transportation may have a strong influence on southern suburbs, and aggravate PM_2.5_ pollution. The northern part of Beijing is surrounded by mountains, and substantial green vegetation may be helpful to cleanse the air [34]. The lower population density in the northern suburbs, together with less human activity, may have contributed to the lower PM_2.5_ concentration observed.

PM_2.5_ concentration shows great seasonal variations, with the most severe PM_2.5_ pollution in winter [12, 31]. Beijing has a northern temperate continental monsoon climate. The official residential heating season is from November to March. The elevated PM_2.5_ level in winter is mainly from coal combustion and biomass burning for residential heating, as in the other northern cities in China [12, 32, 35]. Years ago, sandstorms were a serious problem, and usually reached Beijing in the spring. These storms involved long-range transport of desert dust, with mineral dust comprising 18.6% of PM_2.5_ mass [16, 31]. However, there is no evidence indicating severe PM_2.5_ pollution in spring. This may be as a result of the implemented Beijing and Tianjin Sandstorm Source Control Project which was set up in 2000 [36].

Regarding the weekly pattern, some studies found that air pollutant concentrations revealed a general weekend effect, with higher levels during the weekdays and lower ones during weekends [37, 38]. However, this pattern does not prevail in all cities, especially for PM_2.5_ [39, 40]. Our results confirm no weekday/weekend difference for PM_2.5_ concentrations in Beijing. Vehicle restrictions on weekdays may be an important explanation of this phenomenon. However, there were obvious periodic oscillations for PM_2.5_, with 2–6 episodes each month. Because pronounced day to day variation of PM_2.5_ may be less influenced by traffic intensity, which is uniform across weekdays and weekends, the concentration fluctuation may be largely caused by meteorological conditions such as solar radiation, formation of convectively mixed boundary layers, and wind [41, 42]. This is somehow also supported by a negative correlation between sunlight hours and PM_2.5_ concentration, as well as a negative correlation between that concentration and wind speed (Fig 6). Furthermore, there are more episodes in winter and fewer in summer. Such variation is possibly due in part to seasonal variations of the air pollutant emission and the atmospheric boundary layer height.

Diurnal PM_2.5_ variations were observed with one or two peaks in each month, similar to the results of other studies [39, 41, 43, 44] (Fig 5). The diurnal variations are dominated by the diurnal cycle of source emissions and the boundary layer height [44]. Generally, the forenoon peak is attributable to enhanced anthropogenic activity during morning rush hour, and decreasing PM_2.5_ in the afternoon is mainly due to the developing boundary layer height, which provides a large volume for PM_2.5_ dilution. Finally, a reduced boundary layer height with increased anthropogenic activity during the afternoon rush hour produces the early nighttime peak. In addition, the PM_2.5_ diurnal variations vary by months. In the colder months (October to January), there are more coal combustion and biomass burning for residential heating, and boundary layer height generally decreases early in the afternoon because of less solar radiation, resulting in higher levels of PM_2.5_ in early nighttime [45, 46].

PM_2.5_ levels in Beijing were strongly correlated for all station pairs (*r*_*s*_ > 0.70), and distance was a powerful predictor of correlation [24]. However, 43.03% of COD values calculated for station-pairs’ daily average concentrations of PM_2.5_ were > 0.20, and those values were positively associated with distance, giving an approximate indication of spatial heterogeneity [47, 48]. This finding suggests that despite strong correlation among the stations, averaging PM_2.5_ concentrations at multiple monitoring stations in Beijing may produce misclassification errors in epidemiological research (e.g., time-series epidemiologic studies evaluating relationships between PM_2.5_ and health events).

Although the influence of meteorological conditions on the diffusion, dilution and accumulation of air pollutants has been widely recognized, it remains inconsistent when considering specific meteorological effects on PM_2.5_ concentration. Previous studies have developed various meteorological predictive models for PM_2.5_, with greater predictive powers judged by adjusted *R* ^2^ (0.79) or cross-variation *R* ^2^ (0.77) [49, 50]. Although model performance remains strong, the predictive ability of our model for PM_2.5_ (adjusted *R* ^2^ = 0.59) was somewhat lower. The difference may be attributed to the use of additional selection, such as land use information [49, 50]. The reason why it cannot be explained fully by meteorological factors may be the complex and diverse human activities related to PM_2.5_.

Among meteorological factors, most studies focused on wind speed, indicating that wind speed is negatively correlated with PM_2.5_ [20, 21, 50–53], and this was also evident in this study. The lag effect of wind speed has also been considered in our study, and the result suggests that PM_2.5_ is affected principally by prior day wind speed. For precipitation, our study is also comparable to the other studies [50, 54]. Fig 6 shows that PM_2.5_ concentration is nearly 10% lower on days with precipitation, compared with those days of no precipitation, owing to the fact that precipitation has a scavenging effect on air pollutants [54, 55].

The results about relative humidity on PM_2.5_ pollution were not consistent. Using correlation analysis, some studies found that the relationship between relative humidity and PM_2.5_ is negative or varies with seasons [20, 51–53]. After controlling for temporal tendency, our results showed that relative humidity is positively correlated with PM_2.5_ according to the GAMM method [21]. The main reason could be that during high relative humidity, there is increased formation of secondary PM with large amounts of gas-phase chemical pollutants (CO, O_3_, SO_2_, and NO_x_) [19, 56]. Such situations are also not conducive to air pollutant diffusion.

There have been few studies exploring the relationship between PM_2.5_ and air pressure, as well as sunlight hours. Our results showed that air pressure has a delayed influence on PM_2.5_ concentration, with a positive correlation. In general, certain weather conditions (e.g. precipitation) following low pressure environment may explain this phenomenon. However, evidence is insufficient and more quantitative research is needed to construct a detailed picture of the impact of air pressure on PM_2.5_ concentration. There is a negative relationship between sunlight hours and PM_2.5_, which may be attributed to a larger atmospheric volume for dilution through an increase in boundary layer height [57].

There are limitations in this study. The sampling stations in the study are not equally distributed and are sparse in some districts, and hence a better designed sampling method should be used in future studies. Furthermore, meteorological factors may have a long-term influence on PM_2.5_. We selected only factors that had strong correlation with PM _2.5_ for modeling. We therefore call for future studies in Beijing to investigate the complicated relationship between PM_2.5_ and meteorological conditions over longer period.

## Conclusions

This study provides baseline information for a comprehensive understanding of the current PM_2.5_ pollution in Beijing. The results indicate that PM_2.5_ concentration has strong spatiotemporal variations. PM_2.5_ pollution is more severe in winter and decreased from the south to the north part of the city. Day to day variations of PM_2.5_ show a long-term trend of fluctuations with 2–6 peaks in each month. Diurnal PM_2.5_ variations are observed, with peaks in the forenoon or early nighttime, or both. There is spatial heterogeneity across the observing stations in Beijing. Meteorological factors influence PM_2.5_ concentration in particular forms. Generally, prior day wind speed, sunlight hours and precipitation are negatively correlated with PM_2.5_, whereas relative humidity and air pressure 3 days earlier are positively correlated with PM_2.5_.

## Supporting Information

S1 Database Data of daily PM_2.5_ concentrations and meteorological variables. (XLSX)

S2 Database Data of hourly PM_2.5_ concentrations. (XLSX)

S1 Table Correlation coefficient matrix of PM_2.5_ and meteorological factors. (XLSX)
